# Expression and Functional Analysis of Storage Protein 2 in the Silkworm, *Bombyx mori*

**DOI:** 10.1155/2013/145450

**Published:** 2013-03-26

**Authors:** Wei Yu, Meihui Wang, Hanming Zhang, Yanping Quan, Yaozhou Zhang

**Affiliations:** ^1^Institute of Biochemistry, College of Life Sciences, Zhejiang Sci-Tech University, Hangzhou, Zhejiang 310018, China; ^2^Zhejiang Provincial Key Laboratory of Silkworm Bioreactor and Biomedicine, Hangzhou, Zhejiang 310018, China

## Abstract

Storage protein 2 (SP2) not only is an important source of energy for the growth and development of silkworm but also has inhibitory effects on cell apoptosis. Endothelial cell (EC) apoptosis is an important contributing factor in the development of atherosclerosis; therefore, study of the antiapoptotic activity of SP2 on ECs provides information related to the treatment of atherosclerosis and other cardiovascular diseases. In this study, the *sp2* gene was cloned and expressed in *Escherichia coli* to produce a 6xHis-tagged fusion protein, which was then used to generate a polyclonal antibody. Western blot results revealed that SP2 levels were higher in the pupal stage and hemolymph of fifth-instar larvae but low in the egg and adult stages. Subcellular localization results showed that SP2 is located mainly on the cell membrane. In addition, a Bac-to-Bac system was used to construct a recombinant baculovirus for SP2 expression. The purified SP2 was then added to a culture medium for human umbilical vein ECs (HUVECs), which were exposed to staurosporine. A cell viability assay demonstrated that SP2 could significantly enhance the viability of HUVEC. Furthermore, both ELISA and flow cytometry results indicated that SP2 has anti-apoptotic effects on staurosporine-induced HUVEC apoptosis.

## 1. Introduction

The vascular endothelium provides a cellular interface between the circulating blood and the vascular smooth muscle of the blood vessel walls. It also has an important role in maintaining the balance of the endovascular environment [1]. Many factors, such as peroxide, ox-low density lipoprotein (LDL), angiotensin I, and tumor-necrosis-factor-(TNF-) *α* can induce the production of reactive oxygen species (ROS) by NADPH oxidase in endothelium and vascular smooth muscle [2], which results in oxidative stress to the endothelial cells (ECs) and, thus, the induction of cell apoptosis. EC dysfunction is a trigger factor for the development of atherosclerosis and other cardiovascular diseases [3]. Many studies have shown that EC apoptosis is one of several atherogenic factors [4, 5].

Additional studies have also shown that hemolymph from the silkworm (*Bombyx mori*) can inhibit insect and mammalian cell apoptosis that is induced by viruses and several chemical inducers, such as staurosporine, camptothecin, and actinomycin D [6–8]. As one such antiapoptotic component in silkworm hemolymph, the 30 K protein has been studied widely [6, 7]. A recent study showed that another protein in silkworm hemolymph, storage protein 2 (SP2), can also inhibit staurosporine-induced HeLa cell apoptosis and ROS generation [8]. Owing to the influence of juvenile hormone and ecdysone, SP2 is synthesized by the fat body of feeding larvae and released into the hemolymph [9]. At the end of the feeding period, it is selectively reabsorbed by the fat body cells [10]. SP2 is presumed to be used as a store of the amino acids required for the development of adult tissues [11]. 

In the current study, we cloned and expressed the gene encoding silkworm SP2 (*sp2*) both in a prokaryotic expression system and a silkworm baculovirus system. The expressed protein in* Escherichia coli* was used to generate polyclonal antibodies. The distribution of SP2 in different developmental stages and different tissues was detected by Western blot. In addition, the expressed protein in the silkworm baculovirus system was used to study the anti-apoptotic effects of SP2 on human umbilical vein ECs (HUVECs) induced by peroxidation. This work will lay a foundation for the development and utilization of protein drugs from economically important insects for treatment of vascular diseases.

## 2. Materials and Methods

### 2.1. Strains, Cell Lines, Animals, and Reagents

*Escherichia coli* strains TG1 and BL21 (DE3) were grown at 37°C in LB medium. The pET-28a(+) expression vector and pFastBac HTB vector were conserved in our laboratory. The silkworm-derived cell line BmN was maintained at 27°C in TC-100 medium (Gibco, USA) supplemented with 10% fetal bovine serum (FBS). *B. mori *nuclear polyhedrosis virus (bacmid, conserved by our lab) was propagated in BmN cells. HUVECs were purchased from the American Type Culture Collection (Manassas, VA, USA) and cultured in Dulbecco's modified Eagle medium (Gibco, USA) supplemented with 10% FBS (Gibco, USA) in a humidified incubator at 37°C in an atmosphere of 5% CO_2_. Fifth-instar silkworm larvae (Jingsong × Haoyue, Showa) were reared on fresh mulberry leaves at 25°C. Male New Zealand rabbits were purchased from the Animal Research Center of Hangzhou Normal University. A DNA gel purification kit and Cy3-labeled goat anti-rabbit IgG were purchased from Promega (Madison, USA). HRP-labeled goat anti-rabbit IgG was purchased from Dingguo Biotechnology (Beijing, China). Cellfectin II Reagent and a Ni-NTA Purification System were sourced from Invitrogen (Carlsbad, USA); a Cell Death Detection ELISA kit was sourced from Roche Diagnostics (Castle Hill, Australia); the Annexin V-FITC/PI apoptosis detection kit was purchased from Invitrogen (Carlsbad, USA); staurosporine was sourced from the Beyotime Company (Shanghai, China). Other reagents were locally purchased products of analytical grade. 

### 2.2. Bioinformatics Analyses

Similarity analyses for the nucleotide and protein sequences were carried out using GenBank BLASTn and BLASTp algorithms. The hydrophobicity analysis and the isoelectric point prediction were conducted on the ExPASy website (http://www.expasy.org/). Simulation for the protein tertiary structure was generated by using the SWISS-MODEL program (http://swissmodel.expasy.org/).

### 2.3. Preparation of Polyclonal Antibodies

The open reading frame (ORF) of* sp2 *was amplified by PCR using the cDNA library of silkworm pupa constructed by our laboratory as a template. The forward and reverse primers were as follows: P1, 5′-CGCGGATCCATGAAGTCTGTCTTAATT-3′; and P2, 5′-CCGCTCGAGTTAATTTTTTGGAACAAC-3′, which contained *Bam*HI and *Xho*I restriction sites, respectively. The *sp2* gene was subcloned into the expression vector pET-28a(+). *Escherichia coli *BL21 (DE3) was transformed with the recombinant plasmid and cultured in LB media, that included 50 *μ*g/mL kanamycin, at 37°C until OD_600_ reached approximately 0.5. Recombinant protein expression was induced with 1 mmol/L IPTG for 4 h. The His-tagged fusion protein was extracted from the bacteria and purified by Ni^2+^-affinity chromatography. The purified protein was subsequently concentrated and desalted by dialysis. The protein content was analyzed following the method of Bradford and then used to immunize the male New Zealand rabbits to prepare the polyclonal antibodies. An ELISA was utilized to determine the polyclonal antibody titer, and the specificity of the polyclonal antibody was detected by Western blot analysis.

### 2.4. Western Blot Assay for the Distribution of SP2

Western blot analysis was used to evaluate the distribution of SP2 in different tissues of the fifth-instar larvae and other developmental stages of the silkworm. Tissues from fifth-instar larvae (head, epidermis, hemolymph, fat body, silk gland, trachea, the Malpighian Tubule, and midgut) were isolated and pulverized into powder in liquid nitrogen. The powdered tissues were then resuspended in a lysis buffer (50 mM Tris-Cl, pH 8.0; 0.15 M NaCl; 5 mM EDTA; 0.5% NP-40; 1 mM dithiothreitol; 5 mg/mL sodium deoxycholate; 100 mg/L PMSF; 5 *μ*g/mL aprotinin, Sigma), and the mixture was incubated on ice for 30 min. The homogenates were centrifuged at 12,000 ×g for 15 minutes at 4°C. The supernatants containing total proteins were equalized by assaying the protein content (following the method of Bradford). Samples were verified by running on a 12% SDS-PAGE and electrophoretically transferred onto polyvinylidene difluoride (PVDF) membranes. The membranes were blocked with 3% skim milk in phosphate-buffered saline (PBS) at 4°C overnight, and rabbit anti-SP2 antibody was added and the solution incubated at room temperature for 2 h. After washing with PBST (PBS + 0.05% Tween-20), the bound antibodies were detected by anti-rabbit IgG followed by a DAB detection system. 

### 2.5. Subcellular Localization of SP2 in BmN Cells

BmN cells were cultured overnight on a glass coverslip that could be used specifically for confocal microscopy. After removing the culture medium, the cells were washed three times for 5 min in PBS and fixed in 4% polyformaldehyde in PBS (pH 7.4) at room temperature for 15 min, followed by permeabilization with 0.2% Triton X-100/PBS for 10 min. The fixed cells were preblocked in 3% BSA in PBS at 4°C for 4 h, followed by incubation with the anti-SP2 polyclonal antibody (diluted 1 : 1000 in blocking buffer) at room temperature for 2 h; cells were incubated simultaneously with control serum, which was obtained from the rabbits before immunization with the antigen. After three 10 min PBST washes, cells were incubated with Cy3-labeled goat anti-rabbit IgG (diluted 1 : 1000, Promega) at 37°C for 2 h and washed twice in PBST. The cells were then incubated with 4–6-diamidino-2-phenylindole (1 g/mL in PBS) at 37°C for 30 min. After the cells were washed once with PBST, they were photographed on a Nikon Eclipse TE 2000-E Confocal Microscope (Nikon, Japan). Images were analyzed using EZ-C1 software (Nikon, Japan). 

### 2.6. Expression of SP2 in a Bac-to-Bac System

The* sp2 *gene was inserted into the MCS of the transfer plasmid pFastBac-HTB between *Bam*H I and *Xho* I sites and transformed into DH10Bac cells. The* sp2 *gene was then transferred into a wild type bacmid (wtbacmid) DNA by homologous recombination to construct the recombinant baculovirus bacmid-*sp2*. After white-blue plaque selection, the positive colonies were selected and analyzed by PCR with M13 universal primers, *sp2 *forward and reverse primers. The recombinant bacmid was transfected into BmN cells for amplification. The third-generation virus (MOI = 10 pfu/cell) was further used to infect BmN cells (1 × 10^6^ cells/flask) for subsequent protein expression. After cultivation for 48 h, the cells were harvested by phosphate buffer solution (PBS, pH 7.4) washes, pulsed sonication and centrifugation at 12,000 rpm for 10 min. The supernatant was subjected to Ni^2+^-affinity chromatography according to the manufacturer's instructions (Invitrogen). SDS-PAGE and Western blot were used to detect the purified fusion protein. The wtbacmid infected BmN cells were used as a control. After purification by affinity chromatography, the SP2 protein was concentrated, and imidazole was removed by dialysis against 25 mM HEPES [4-(2-hydroxyethyl) piperazine-1-ethanesulfonic acid), Sigma]; pH 7.5, 100 mM NaCl. The protein content was analyzed by following the method of Bradford.

### 2.7. Induction of HUVEC Apoptosis

HUVECs were isolated by 0.25% trypsinization and cultured in Dulbecco's modified Eagle medium (Gibco) supplemented with 10% FBS and maintained in a humidified atmosphere with 5% CO_2_ at 37°C air until 70–80% confluence was reached. Cells (from the experimental group) were pretreated for 24 h with culture medium containing purified SP2 protein (at a final concentration of 0.5, 1, and 2 *μ*g/mL). Negative and normal controls did not receive any intervention. After washing twice with Hank's balanced salt solution (Gibco), the experimental and negative groups were both exposed to 2 mL STS (1 *μ*M) for 2 h to induce apoptosis, whereas the normal group was treated with 2 mL Hank's balanced salt solution. The experimental group was then washed twice with Hank's balanced salt solution and cultured in culture medium in the presence of purified SP2 protein (at a final concentration of 0.5, 1, and 2 *μ*g/mL). HUVEC viability and apoptosis were evaluated after 12 h.

### 2.8. Cell Viability Analysis Using an MTT Assay

 Cell viability was estimated using MTT (3-[4,5-dimethylthiazol-2-yl]-2,5-diphenyl tetrazolium bromide; Sigma) assay. Cells were seeded into 96-well plates at densities of 1 × 10^3^cells/well and incubated overnight. Following the treatments as indicated above, 10 *μ*L MTT (5 mg/mL) was added to each well and incubated for 4 h. The insoluble formazan crystals were dissolved in 200 *μ*L/well dimethylsulfoxide, and absorbance was measured at 490 nm to calculate the relative cell viability ratio. Each experimental condition was repeated at least three times.

### 2.9. Detection of DNA Fragmentation

DNA fragmentation was evaluated by histone-associated DNA fragments using a photometric enzyme immunoassay (Cell Death Detection ELISA, Roche), according to the manufacturer's instructions. Each experimental condition was repeated at least three times.

### 2.10. Flow Cytometric Analysis

Cells at a density of 1 × 10^6^ cells/mL were harvested and washed twice with ice-cold PBS. For the quantitative assessment of apoptosis, staining with Annexin V-FITC and PI labeling was undertaken before flow cytometric analysis, according to the manufacturer's recommendations (Annexin V-FITC/PI apoptosis detection kit, Invitrogen). FACS analysis was accomplished using a FACSCalibur (Becton Dickinson, San Jose, CA, USA).

### 2.11. Statistical Analysis

The data were expressed as mean ± SEM and analyzed by the Student's *t*-test and the Newman-Keuls test. Differences with a value of *P* < 0.05 were considered statistically significant.

## 3. Results

### 3.1. Bioinformatics Analysis

**Figure 1:**
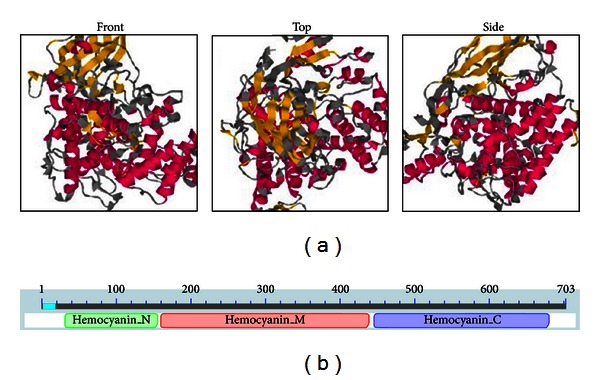
The tertiary structure prediction (a) and three conserved domains (b) of silkworm SP2 protein. (a) Coloring is defined by the dss rasmol script, to show predicted secondary structure (red: helix; yellow: strand; gray: other); (b) Three conservative structural domains of SP2: hemocyanin_N domain (amino acids 33–157), hemocyanin_M domain (amino acids 161–442), and hemocyanin_C domain (amino acids 446–680).

Sequence analysis revealed that the ORF of *sp2 *(accession number DQ443358) was 2112 bp in length and encoded a protein of 703 amino acids, with a predicted molecular mass of 83.45 kD and a theoretical isoelectric point of 5.7. Through BLASTP comparison, three conservative structural domains of the hemocyanin superfamily were discovered: hemocyanin_N domain, hemocyanin_M domain, and hemocyanin_C domain. Hemocyanin can be any of a group of copper-containing respiratory proteins that serve an oxygen-carrying function in the blood of some arthropods and most mollusks [12]. The tertiary structure of silkworm SP2 was predicted using SWISS-MODEL (Figure 1).

### 3.2. Preparation of Silkworm SP2 Polyclonal Antibodies

**Figure 2:**
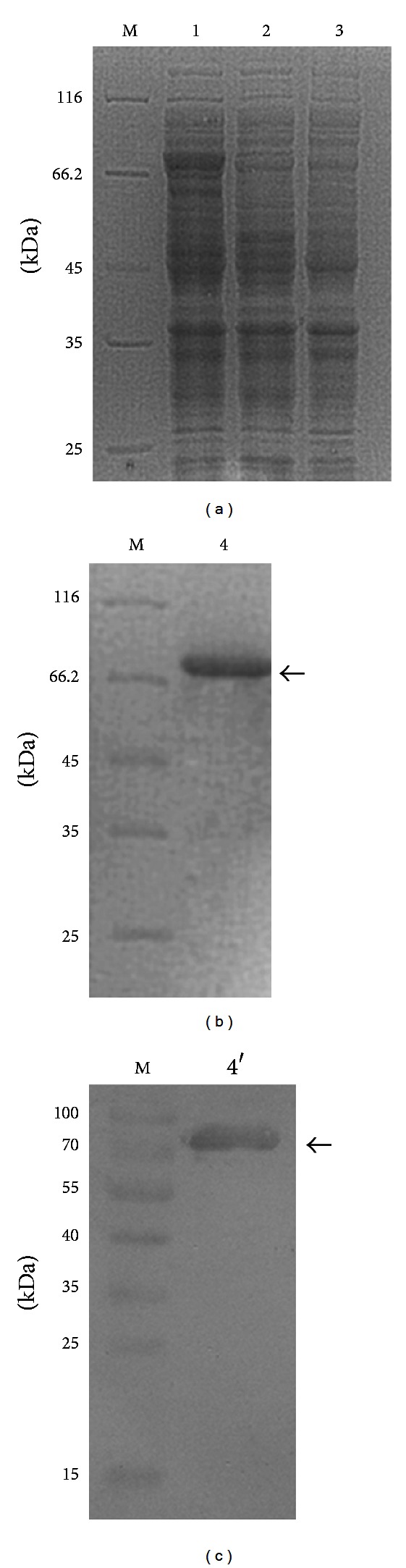
Expression (a), purification (b), and Western blot analysis (c) of the SP2 protein expressed in *Escherichia coli*. M: protein molecular weight marker; 1: recombinant bacteria after inducing; 2: recombinant bacteria before inducing; 3: empty vector after inducing; 4: purified of the fusion protein; 4′: Western blot analysis.

The ORF for *sp2* was subcloned into the prokaryotic expression vector pET-28a (+). The His-tagged fusion protein was expressed in *E. coli *BL21 (DE3) (Figure 2(a)) and purified by Ni^2+^-affinity chromatography. The purified His-SP2 fusion protein was successfully detected by SDS-PAGE (Figure 2(b)). The predicted molecular weight of the fusion protein, including a 3.56-kD His-tag, was 87 kD, which is concordant with the calculated value. The concentration of purified SP2 protein was determined by use of the Bradford assay to be 1.5 mg/mL. With the affinity-purified proteins, anti-SP2 polyclonal antibodies were generated by immunizing a male New Zealand rabbit. The titer of the polyclonal antibody, as determined by ELISA, was 1 : 6400. Western blot analysis indicated that the antibody reacted specifically with purified His-SP2 fusion protein (Figure 2(c)).

### 3.3. Expression Analysis of SP2 in Silkworm

**Figure 3:**
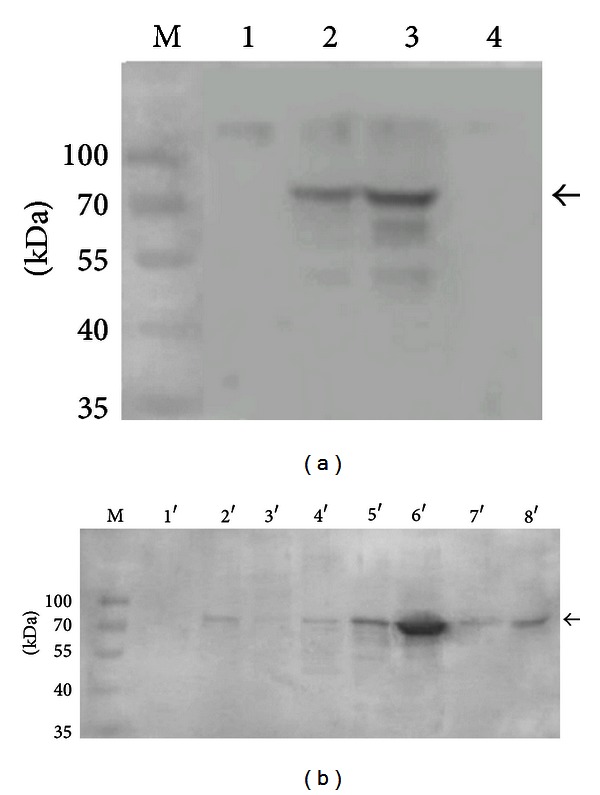
The expression level of *sp2 *in the developmental stages of the silkworm *Bombyx mori* (a) and in different tissues of fifth-instar *B. mori* larva (b). 1: egg; 2: larva; 3: pupa; 4: adult; 1′: midgut; 2′: the Malpighian tubule; 3′: trachea; 4′: silk gland; 5′: fat body; 6′: hemolymph; 7′: epidermis; 8′: head. The arrow indicates the SP2 protein.

To determine the distribution of SP2 in different tissues of the fifth-instar larva and other development stages of the silkworm, Western blot analysis was performed on protein extracts. The results showed that the level of SP2 was very high in the pupal and larval stages but extremely low in the egg and adult stages (Figure 3(a)). In the fifth instar larvae, the SP2 level was highest in the hemolymph and fat body and lowest in the trachea and midgut (Figure 3(b)).

### 3.4. Subcellular Localization of SP2

**Figure 4:**
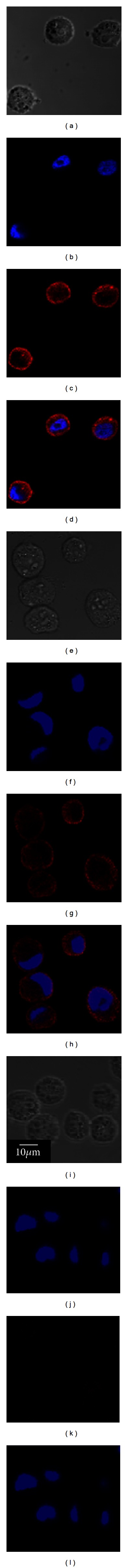
Subcellular localization of SP2 detected using Cy3-labeled goat anti-rabbit IgG and DAPI. ((a), (e), and (i)) Cells at the transmission light; ((b), (f), and (j)) nucleolus dyed with DAPI; ((c), (g), and (k)) SP2 dyed with Cy-3; ((d), (h), and (l)) merged images; ((i), (j), (k), and (l)) negative control group, using control rabbit serum.

The treated cells were examined under a Nikon ECLIPSE TE2000-E Confocal Microscope, and images were analyzed using EZ-C1 software. DAPI-stained nuclei fluoresce red when stimulated with 353 nm light, and Cy3-labeled goat anti-rabbit IgG fluoresces red when stimulated with 550 nm light. Our results indicated that SP2 is mainly located in the cell membrane and only partly in the cytoplasm (Figure 4).

### 3.5. Expression and Purification of SP2 in a Bac-to-Bac System

**Figure 5:**
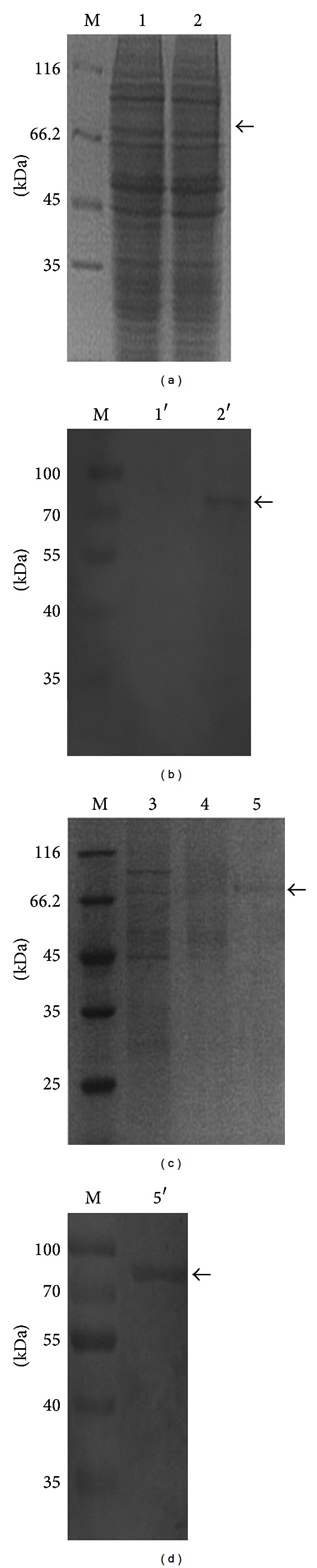
SDS-PAGE and Western blot analysis of SP2 protein expressed in BmN cells. (a) SDS-PAGE analysis of BmN cells lysates; (b) Western blot analysis of BmN cells lysates; (c) SDS-PAGE analysis of gradient purified SP2 protein; (d) Western blot analysis of purified SP2 protein; M: protein molecular weight marker; 1, 1′: total protein of BmN cells infected by wtbacmid; 2, 2′: total protein of the BmN cells infected by vBm-*sp2*; 3: protein that discharges out from the column; 4: eluted protein with 20 mM imidazole; 5: eluted protein with 250 mM imidazole; 5′: Western blot analysis of the purified fusion protein.

To study the function of SP2 protein, recombinant bacmid-*sp2* was used to infect BmN cells for expression of the His-tagged protein, and wtbacmid was used as a control (Figure 5(a)). Owing to the low expression level of natural SP2 protein in BmN cells, the results of Western blot did not show the specific band in the BmN cells lysates infected by wtbacmid (Figure 5(b)). The BmN cell lysates were subjected to Ni^2+^-affinity chromatography. The concentration of purified SP2 protein was determined by use of the Bradford assay to be 0.108 mg/mL. The results of SDS-PAGE and Western blot analysis indicated that the purified fusion protein SP2 had a molecular mass of approximately 87 kD (Figures 5(c) and 5(d)).

### 3.6. STS-Induced HUVEC Apoptosis and Apoptosis Assay

STS triggers within the cell both morphological changes and internucleosomal DNA fragmentation, which represents apoptosis [13]. An increasing concentration of STS was applied to examine the effects on HUVECs apoptosis. Apoptotic cells accompanying DNA fragmentation were analyzed at 24 h after STS treatment using ELISA. A low concentration (0.1 *μ*M) of STS did not affect apoptosis, whereas a high concentration (2 *μ*M) induced necrosis, and 1 *μ*M triggered DNA fragmentation efficiently compared with the positive control (camptothecin) provided by the kit. Based on these data, the following experiments were examined using 1 *μ*M STS.

**Figure 6:**
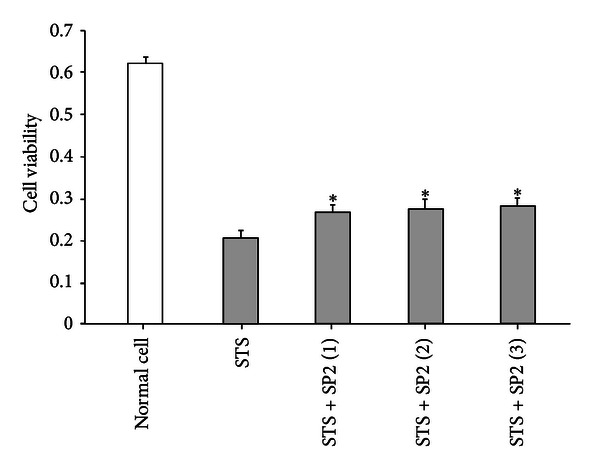
The effect of SP2 on the viability of HUVEC. The vertical axis is the 490 nm absorbance; STS is the negative control; STS + SP2 (1–3) are the treatment groups, in which the final concentrations of the SP2 sample were 0.5 *μ*g/mL, 1 *μ*g/mL, and 2 *μ*g/mL, respectively. Data are mean ± SEM, *n* = 3. *Indicates values significantly different from negative control using Student's *t*-test; *P* < 0.05.

**Figure 7:**
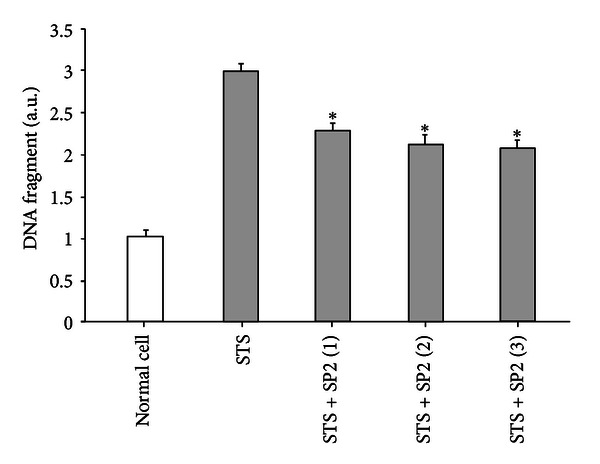
The effects of SP2 on DNA fragmentation caused by STS-induced apoptosis of HUVEC. The vertical axis is the DNA fragment unit; STS is the negative control; STS + SP2 (1–3) are the treatment groups, in which the final concentrations of SP2 sample were 0.5 *μ*g/mL, 1 *μ*g/mL, and 2 *μ*g/mL, respectively. Values are expressed as mean ± SEM (*n* = 3). *Indicates values significantly different from negative control using Student's *t*-test; *P* < 0.05.

**Figure 8:**
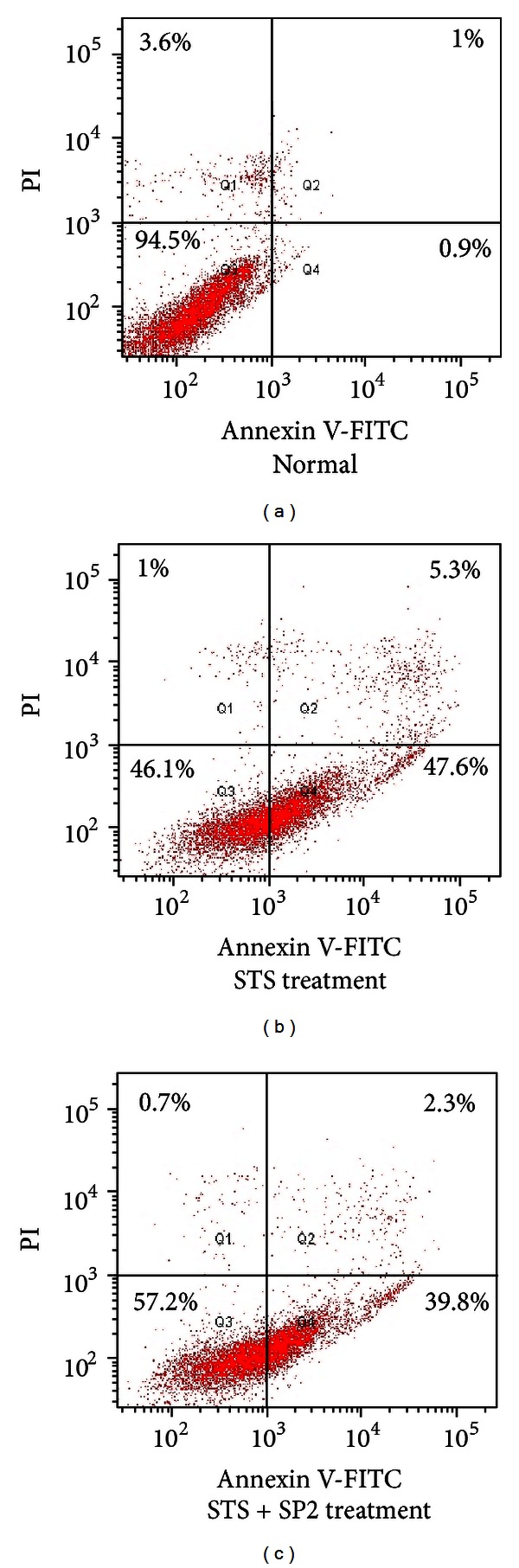
Detection of the antiapoptotic effect of SP2 by FCM. The flow cytometry profile represents Annexin V-FITC staining on *x*-axis and PI on *y*-axis. The number represents the percentage of early apoptotic cells in each condition (lower right quadrant). The cells in the upper right quadrant indicate Annexin-positive/PI-positive, late apoptotic cells. (a) Normal cell group; (b) STS treatment group; (c) STS + SP2 (1 *μ*g/mL) treatment group; damaged (Annexin V^−^/PI^+^), apoptotic (Annexin V^+^/PI^−^), and vital (Annexin V^−^/PI^−^) cells are shown in the Q1, Q2/Q4, and Q3 regions, respectively.

Cell viability was ameliorated by addition of the purified recombinant SP2 protein in a dose-dependent manner (Figure 6), indicating that SP2 can enhance apoptotic HUVEC viability. To investigate the inhibitory effect of SP2 protein on STS-induced HUVEC apoptosis, an ELISA was used to detect the DNA fragmentation. As shown in Figure 7, HUVEC apoptosis as measured by DNA fragmentation was significantly attenuated by recombinant SP2 protein in a dose-related manner. To quantify the apoptotic HUVECs, an Annexin V-FITC/PI apoptosis detection kit was used and assessed by flow cytometry. Apoptotic cells were identified by fluorescein (FITC) conjugated to the human anticoagulant-Annexin V, which was able to bind to phosphatidyl serine (PS) in the outer leaflet of the membrane of apoptotic cells. To distinguish cells that had lost membrane integrity, propidium iodide (PI) was added before analysis. As shown in Figure 8, the apoptosis rate of untreated HUVECs (normal control) was 1.9%, whereas the percentage of apoptotic HUVECs (negative control) that had been treated with 1*μ*M STS was 52.9%. Compared with the negative control, the apoptosis rate of STS + SP2 (1 *μ*g/mL)-treated HUVECs decreased to 42.1%. These results indicate that SP2 has anti-apoptotic effects on HUVEC apoptosis induced by STS.

## 4. Discussion

As the major regulator of vascular homeostasis, the endothelium exerts several vasoprotective effects, such as vasodilation, suppression of smooth muscle cell growth, and inhibition of inflammatory responses [14]. EC apoptosis has an important role in the diseased state of atherosclerosis [15]. A large body of evidence has suggested that endothelial dysfunction is caused by superoxide and other ROS [16, 17]. Supplementation with antioxidant vitamins C and E can restore endothelial function and, therefore, retard the progression of atherosclerosis [18]. Other factors, such as vascular endothelial growth factor (VEGF), low concentration of nitric oxide (NO) in serum, calcium antagonists, prostacyclin, and estrogen, can also inhibit EC apoptosis [19].

Many clinical trials have been done to investigate ways of treating diseases associated with apoptosis with anti-apoptotic genes and proteins [20, 21]. Silkworm hemolymph and its 30 K protein have been reported to exhibit anti-apoptotic activity in various mammalian and insect cell systems [7] and could have therapeutic potential in diseases related to apoptosis. In the current study, another potential anti-apoptotic protein in silkworm hemolymph (SP2) was studied. As a member of the hemocyanin superfamily, the SP2 protein has multiple biological functions [8–11]. Hemocyanins are large copper-containing proteins that transport oxygen in the hemolymph of many arthropod and mollusk species [22]. Most hexamerins are considered to be storage proteins, providing energy, serving as carrier protein or possibly involved in the humoral immune response. Therefore, they are considered to be an important immune molecule in arthropod [23]. Owing to the important role of hexamerins during arthropod development, we speculated that silkworm SP2 protein might also have an important role during cell apoptosis. To explore its functions, the SP2 protein was expressed and purified both in a prokaryotic expression system and silkworm baculovirus system. The purified SP2 prokaryotic expression product was used to generate monoclonal antibodies to study the distribution and location of SP2 in silkworm, whereas the purified expression product was used to study its antiapoptotic function.

Our analysis of the level of SP2 in different tissues of the fifth-instar larva and other developmental stages of the silkworm showed that SP2 levels were highest in the hemolymph and fat body and lowest in the egg and adult stages. These results suggest that* sp2* gene activity is affected by juvenile and molting hormones [24]. From the analysis of the subcellular localization of SP2, we found that, in the BmN cell line, SP2 was localized to both the cell membrane and cytoplasm but was found primarily in the cell membrane. Because SP2 is a secreted protein and has a signal peptide predicted by bioinformation (data not shown), our results suggest that it is translocated across the cell membrane guided by the signal peptide.

To study the anti-apoptotic activity of SP2 on vascular EC apoptosis, we constructed an STS-induced HUVEC apoptosis model. The STS treatment was able to induce generation of ROS, which results in oxidative stress to the cells. STS at a concentration of 1 *μ*M exerted prominent apoptotic effects in cultured HUVEC. An MTT assay, DNA fragmentation detection, and flow cytometric analysis showed that the purified recombinant SP2 protein could significantly enhance the viability of HUVEC and inhibit HUVEC apoptosis induced by STS. These findings provide new insights into the prevention of endothelial dysfunction and could ultimately provide therapies for atherosclerosis.
